# Influence mechanisms of health Qigong embodied experience on meaning in life among older adults: a mixed-methods study

**DOI:** 10.3389/fpubh.2026.1871897

**Published:** 2026-09-17

**Authors:** Doudou Yang, Qiong Yao

**Affiliations:** 1 Department of Physical Education, Guangdong University of Science and Technology, Dongguan, China; 2 School of Wushu, Guangzhou Sport University, Guangzhou, China

**Keywords:** embodied cognition theory, health Qigong bodily regulation experience, health Qigong psychological regulation experience, meaning in life, psychological capital

## Abstract

**Objective:**

This study used a mixed-methods approach (qualitative and quantitative) to develop the Health Qigong Embodied Experience Scale, code its dimensions, and examine associations between Health Qigong embodied experience and meaning in life among older adults, with particular attention to the mediating roles of psychological capital (optimism, resilience, hope, and self-efficacy).

**Methods:**

This study first employed thematic analysis to code themes of Health Qigong embodied experience, from which two dimensions were extracted: bodily regulation experience and psychological regulation experience. Next, convenience sampling was used to survey 500 older adults across six communities in Guangzhou. Data were collected using the Health Qigong Bodily Regulation Experience Scale, the Health Qigong Psychological Regulation Experience Scale, the Psychological Capital Scale, and the Meaning in Life Scale. SPSS 27.0 and AMOS 26.0 were used to assess scale reliability and validity and to conduct confirmatory factor analysis (CFA). We then conducted common method bias testing, descriptive statistics, and correlation analyses. Finally, Model 4 of the PROCESS 4.0 macro was applied to examine the mediating roles and the total association hypotheses.

**Results:**

The results indicated that Health Qigong bodily regulation experience and psychological regulation experience were each associated with older adults’ meaning in life (*β* = 0.532, *p* < 0.01; *β* = 0.593, *p* < 0.01). Optimism partially mediated the relationships between bodily regulation experience (*β* = 0.048, Bootstrap 95% CI [0.012, 0.086]) and psychological regulation experience (*β* = 0.045, Bootstrap 95% CI [0.009, 0.082]) with meaning in life. Resilience partially mediated the relationships between bodily regulation experience (*β* = 0.211, Bootstrap 95% CI [0.138, 0.282]) and psychological regulation experience (*β* = 0.205, Bootstrap 95% CI [0.139, 0.269]) with meaning in life. Hope partially mediated each of the relationships between bodily regulation experience (*β* = 0.076, Bootstrap 95% CI [0.028, 0.130]) and psychological regulation experience (*β* = 0.066, Bootstrap 95% CI [0.018, 0.115]) and meaning in life. Notably, self-efficacy did not play a mediating role (*β* = 0.025, Bootstrap 95% CI [−0.046, 0.091]; *β* = 0.028, Bootstrap 95% CI [−0.024, 0.076]).

**Conclusion:**

In the qualitative component, this study developed a new scale. In the quantitative component, conservation of resources (COR) theory was applied at the macro level to frame the structural relationships among the mediation pathways, whereas embodied cognition theory was used at the micro level to explain the mechanisms linking Health Qigong embodied experience to older adults’ meaning in life, alongside psychological capital playing a mediating role. Among these, optimism, hope, and resilience each played a partially mediating role in these relationships, whereas self-efficacy did not. This indicates that, through Health Qigong embodied experience, older adults’ meaning in life is nurtured by process-oriented and acceptance-based psychological resources rather than by outcome-oriented self-efficacy.

## 1 Introduction

With declining marriage and fertility rates, coupled with rising life expectancy, population aging is accelerating worldwide. Projections indicate that the proportion of older adults will reach 16.3% by 2050 and may rise to 21% in the latter half of this century, marking a severe stage of global population aging (1). This trend not only *p*laces considerable strain on global social security systems but also profoundly affects older adults’ physical and mental health. Under the dual pressures of natural physiological decline and social role transitions, many older adults face multiple challenges, including chronic diseases, weakened social connections that lead to a sense of psychological loss, and a diminished sense of meaning in life. Consequently, restoring older adults’ pursuit of meaning in life and alleviating the physical and mental health consequences associated with population aging have become pressing concerns.

Against this backdrop, the mind–body view rooted in embodied cognition theory offers an innovative research framework. This theory transcends traditional mind–body dualism by emphasizing that cognition, emotion, and meaning generation are grounded in the body’s physical properties and experiential activities (2). The body is not merely a vessel or tool for the mind; rather, it serves as the foundation for perceiving the world, forming experiences, and understanding the self. Accordingly, an embodied “body-subjectivity” perspective is needed, one that restores the centrality of the body in individual experience and examines the interactions among body, mind, and behavior in specific contexts, thereby systematically capturing the complex perceptual mechanisms underlying the mind–body connection (3).

Health Qigong is a health-promoting exercise centered on integrating physical movement, breathing regulation, and psychological adjustment, with a deep emphasis on balanced body–mind development. Its theoretical foundations draw on the wellness traditions of Daoism, Buddhism, and traditional Chinese medicine. The practice traces its historical roots to the ancient “dao yin” (guided movement) exercises of the pre-Qin period (before 221 BCE), when practitioners observed natural phenomena and animal behaviors and imitated their motions and breathing rhythms to cultivate health and vitality. Since the early 2000s, the General Administration of Sport of China has systematically standardized Health Qigong, establishing nine official routines including Ba Duan Jin (Eight-section Brocade), Wu Qin Xi (Five-animal Frolics), and Yi Jin Jing (Muscle-tendon Change Classic) (4). As an important component of Chinese intangible cultural heritage, Health Qigong is a highly structured, embodied practice that prioritizes internal experience. Its practice follows the logic of embodied experience. First, at the level of bodily regulation, the close coordination of gentle, slow movements with deep, elongated, and refined breathing allows practitioners to engage in a profound dialogue with their own bodies. This conscious, coordinated practice helps harmonize vital energy (qi) and blood, supporting immune function (5). Second, at the level of psychological regulation, Health Qigong requires practitioners to focus their attention on ongoing movements and breathing while setting aside external distractions, thereby cultivating a state of inner mental focus and calm. This process facilitates emotional balance and alleviates adverse psychological symptoms such as anxiety, helping to rekindle vitality and enable the realization of life’s value (6). These two aspects complement each other, jointly constituting an embodied experience that fosters body–mind integration and opens a promising avenue for improving the physical and mental health of older adults.

A review of the literature documents a substantial body of research on the benefits of Health Qigong. Most findings to date, however, have clustered within disciplines such as medicine (7), communication studies, and the philosophy and culture of sport. These studies have tended to approach Health Qigong either as a positive intervention targeting physiological indicators across diverse populations or as a vehicle for sports culture dissemination and heritage (7, 8). Important as these lines of inquiry are, they have, to some extent, left underexamined the psychological dimensions embedded in Health Qigong as a cultural practice and experiential process, particularly the application of embodied cognition theory to explore, in depth, how the multidimensional embodied experience of Health Qigong bears on older adults’ meaning in life. Accordingly, the present study extends beyond these limitations by applying embodied cognition theory and developing the Health Qigong Embodied Experience Scale. It examines whether the embodied experiences of bodily and psychological regulation that older adults acquire through Health Qigong practice are linked to gains in psychological capital and, in turn, to a strengthened affirmation of their own life value. This line of inquiry enriches understanding of the internal processes connecting bodily activity and mental health, addresses an underexplored area at the intersection of psychology and embodied cognition research on Health Qigong (9), and advances body–mind integration by grounding it in older adults’ felt embodied experience, thereby engaging constructively with the challenges of population aging.

## 2 Theoretical foundations

### 2.1 Health Qigong embodied experience and meaning in life

Meaning in life, defined as individuals’ clear perception and emotional experience of their own existential value and life goals, is a key predictor of mental health and subjective well-being among older adults (10). It reflects people’s comprehension or recognition of the significance of their lives, accompanied by awareness of their life goals, tasks, and purpose, and comprises two dimensions: the presence of meaning and the search for meaning (11). For older adults, the sources of meaning in life often diminish due to social role transitions and changes in physical functioning. Meaning in life is a higher-order psychological construct whose generation and maintenance are inseparable from concrete bodily experiences. From the perspective of embodied cognition theory, bodily experience is not merely an epiphenomenon of cognitive processes but constitutes their very foundation (12). The bodily and psychological regulation experiences afforded by Health Qigong contribute to the construction of meaning in life through two distinct yet complementary pathways: bodily regulation enables practitioners to consistently perceive improvements in physical functioning and the smooth circulation of qi and blood, while psychological regulation fosters inner focus and emotional calm. Through the “body schema” mechanism, these two forms of positive bodily feedback gradually reshape individuals’ pre-reflective perception of their own existential state, such that the body is no longer experienced as a source of aging and decline but becomes a vehicle of agency and vitality (13). It is this existential shift, from a sense of bodily decline to a sense of bodily agency, that enables older adults to reaffirm through embodied experience that they remain capable and that their lives continue to hold value, thereby establishing an irreplaceable embodied foundation for the development of meaning in life.

#### 2.1.1 Bodily regulation experience and meaning in life

The bodily regulation experience of Health Qigong enables practitioners to develop refined perceptions of their physical state, movement processes, and the flow of vital energy (qi) through specific body movements and breathing patterns. According to the concept of the “body schema” in embodied cognition theory, individuals form an implicit cognitive structure concerning their bodily capabilities, boundaries, and possibilities through continuous body–environment interactions (12). This body schema not only guides spatial orientation and motor coordination but also serves as a crucial foundation for meaning in life (14). Breivik (13) further emphasizes that the body is the source of subjectivity and that bodily motor and perceptual abilities are directly associated with individuals’ mode of being in the world and their sense of meaning.

Previous research has shown that practicing Tai Chi for health cultivation can enhance individuals’ meaning in life and improve their mental health (15). Wu et al. (16) found that Tai Chi, Ba Duan Jin, and walking positively affected the mental health of older adults living alone in urban areas. Moreover, with social participation as a mediator and exercise environment as a moderator, these positive mental health outcomes facilitated older adults’ realization of their life value in society. In an experimental study, Health Qigong practitioners demonstrated significantly higher levels of proprioceptive acuity and body awareness than the control group, and body awareness was positively correlated with their meaning in life (8). During Health Qigong practice, older adults perform slow, gentle, continuous movements coordinated with deep, even, and prolonged abdominal breathing while directing their attention toward internal bodily sensations. This enhanced interoceptive awareness is associated with a deeper perception of life’s meaning and value and with the emergence of positive psychological states (9). Furthermore, Health Qigong embodies traditional Chinese health cultivation wisdom, through which older adults experience a profound integration of life with nature and culture during practice, an experience accompanied by a deepening of their understanding and acceptance of the laws of life (17). Taken together, the bodily regulation experience of Health Qigong is linked to the emergence of meaning in life among older adults, alongside strengthened positive perceptions of their own physical functioning.

#### 2.1.2 Psychological regulation experience and meaning in life

Psychological regulation emphasizes achieving tranquility, focus, and relaxation through attentional control and emotional regulation, grounded in coordinated movement and breathing. From the perspectives of extended mind and situated cognition within embodied cognition theory, mental processes are realized primarily through the coupling of the body with specific contexts, and structural features of the environment as well as bodily postures and movements directly influence cognitive and emotional states (18). From a mindfulness perspective, researchers have further shown that body-oriented attentional training (e.g., breath awareness) enables individuals to break automatic thought patterns, shifting from the “doing mode” to the “being mode,” thereby reducing rumination and worry about the past and future while enhancing acceptance of present-moment experience (19). This present-focused psychological state constitutes an important source of meaning in life.

In the psychological regulation practice of Health Qigong, older adults direct their attention to internal bodily sensations through techniques such as “focusing on the navel” and “guiding qi with the mind”. This training is consistent with mindfulness practice (20). Wang et al. (21) used Health Qigong as a mindfulness-based intervention and found that after 6 months of practicing the Six-Character Formula, participants showed significant increases in mindfulness and gradual reductions in anxiety and depression. Previous research has shown that mindfulness training significantly enhances meaning in life (22), with meaning showing particularly consistent gains. For older adults, psychological regulation experiences are linked to acceptance and appreciation of their present mind–body state, to reduced negative psychological symptoms associated with future-oriented worries, and to the experience, through embodied awareness, of the joy and well-being that life affords.

### 2.2 Mediating role of psychological capital: a conservation of resources theory framework

The transformation of Health Qigong embodied experience into meaning in life is not direct; rather, it depends on the accumulation and conversion of individual psychological resources. To account for this mediating mechanism, the present study draws on Conservation of Resources (COR) theory as an overarching theoretical framework.

COR theory holds that individuals are motivated to acquire, maintain, and protect resources, which include both material resources (e.g., health status, physical functioning) and psychological resources (e.g., positive psychological states, personal traits); the acquisition and accumulation of resources constitute a foundational mechanism for mental health and psychological growth (23). A key tenet of COR theory is that resources do not exist in isolation but operate synergistically as “resource caravans,” such that the accumulation of one resource facilitates the growth of others, generating a resource gain spiral.

Within this framework, Health Qigong embodied experience can be understood as a systematic process of resource gain: through bodily regulation and psychological regulation, practitioners continuously accumulate positive embodied experience. These experiential resources, however, do not act directly on meaning in life; instead, they are first consolidated into psychological capital. Psychological capital, a construct introduced by Luthans and colleagues from the perspectives of positive psychology and positive organizational behavior, is defined as a positive psychological state that individuals manifest during growth and development and encompasses four dimensions: self-efficacy, hope, resilience, and optimism (24). It represents the core personal psychological resource identified by COR theory (25). When this reserve is sufficiently enriched, individuals retain the psychological capacity to extract meaning and construct goals from daily experiences even in the face of physical decline and aging, thereby enabling the conversion of Health Qigong embodied experience into meaning in life.

To summarize, within the COR framework, the influence of Health Qigong embodied experience on meaning in life follows a “resource gain (embodied experience) → resource reserve (psychological capital) → resource deployment (meaning in life)” pathway. Psychological capital serves as the key mediator in this sequence, providing a unified explanatory mechanism through which bodily regulation and psychological regulation, two qualitatively distinct forms of embodied experience, contribute to meaning in life.

#### 2.2.1 Mediating role of psychological capital in the relationship between bodily regulation experience and meaning in life

Physical exercise has consistently been identified as an important determinant of psychological capital. Park et al. (26), for instance, demonstrated that higher levels of physical activity were associated with higher levels of psychological capital and that sustained moderate-intensity physical exercise significantly enhanced psychological capital. From the perspective of embodied cognition theory, psychological capital is not an abstract trait but an embodied resource rooted in bodily experience and continuously generated and strengthened through physical action (27). In Health Qigong bodily regulation practice, older adults receive positive feedback on their physical functioning through sustained limb movements and breathing control: a sense of control from completing movements, a sense of energy from deep breathing, and a sense of comfort from physical relaxation. Through neuroendocrine regulation, these positive embodied experiences can gradually internalize into positive psychological resources. Luthans and Youssef (28) showed that physical activity interventions effectively enhanced psychological capital, particularly in older adults, as body-based interventions could simultaneously activate multiple dimensions of psychological capital. Studying the Health Qigong Five-Animal Exercises, Jiang et al. (29) found that practitioners exhibited significant improvements in self-efficacy and hope alongside enhanced physical functioning, and these improvements positively correlated with years of practice. More critically, a longitudinal study by Avey et al. (30) showed that psychological capital significantly mediated the relationship between meaning in life and subjective well-being. Individuals with higher psychological capital are better able to extract meaning and construct goals from daily experiences. Older adults who experience gains in psychological capital linked to bodily regulation maintain hope for the future and continue to pursue their goals even when facing the functional limitations of physical and mental aging, alongside a sustained and deepened experience of meaning in life.

#### 2.2.2 Mediating role of psychological capital in the relationship between psychological regulation experience and meaning in life

As a body-oriented psychological training, psychological regulation experience allows individuals to withdraw attention from external distractions and focus on present-moment bodily sensations. The most direct outcome of this training is an improved emotional state. Fredrickson and Losada (31) empirically demonstrated that mindfulness practices grounded in physical awareness enhanced individuals’ psychological resilience, primarily through positive emotions generated by physical practice. This experience concretely reflected the resilience and optimism dimensions of psychological capital. A survey by Cheng and Tse (32) among older adults found that regular Health Qigong practitioners had significantly higher optimism than non-practitioners, and that optimism positively correlated with their meaning in life. In other words, the more optimistic older adults were, the more they affirmed the value of their own life. Individuals with higher psychological capital are generally more adept at extracting meaning and constructing goals from daily experiences; they are less likely to negate their overall life value due to temporary setbacks (30). For older adults, psychological capital associated with Health Qigong psychological regulation is linked to the psychological resources that support coping with the various challenges of aging and to the continuous experience of fullness and fulfillment in the meaning of life within integrated body–mind practice.

### 2.3 The present study

In summary, drawing on embodied cognition theory, this study investigates the pathways through which the bodily regulation experience and psychological regulation experience of Health Qigong relate to older adults’ meaning in life. A theoretical framework for the mediation pathways is developed through the lens of COR theory, and the mediating roles of the dimensions of psychological capital are examined within this framework. Accordingly, this study constructs the hypothesized research models (see Figures 1, 2) with bodily regulation experience and psychological regulation experience as independent variables, self-efficacy, optimism, resilience, and hope as mediating variables, and meaning in life as the dependent variable. Based on this model, the following hypotheses are proposed:

**Figure 1:**
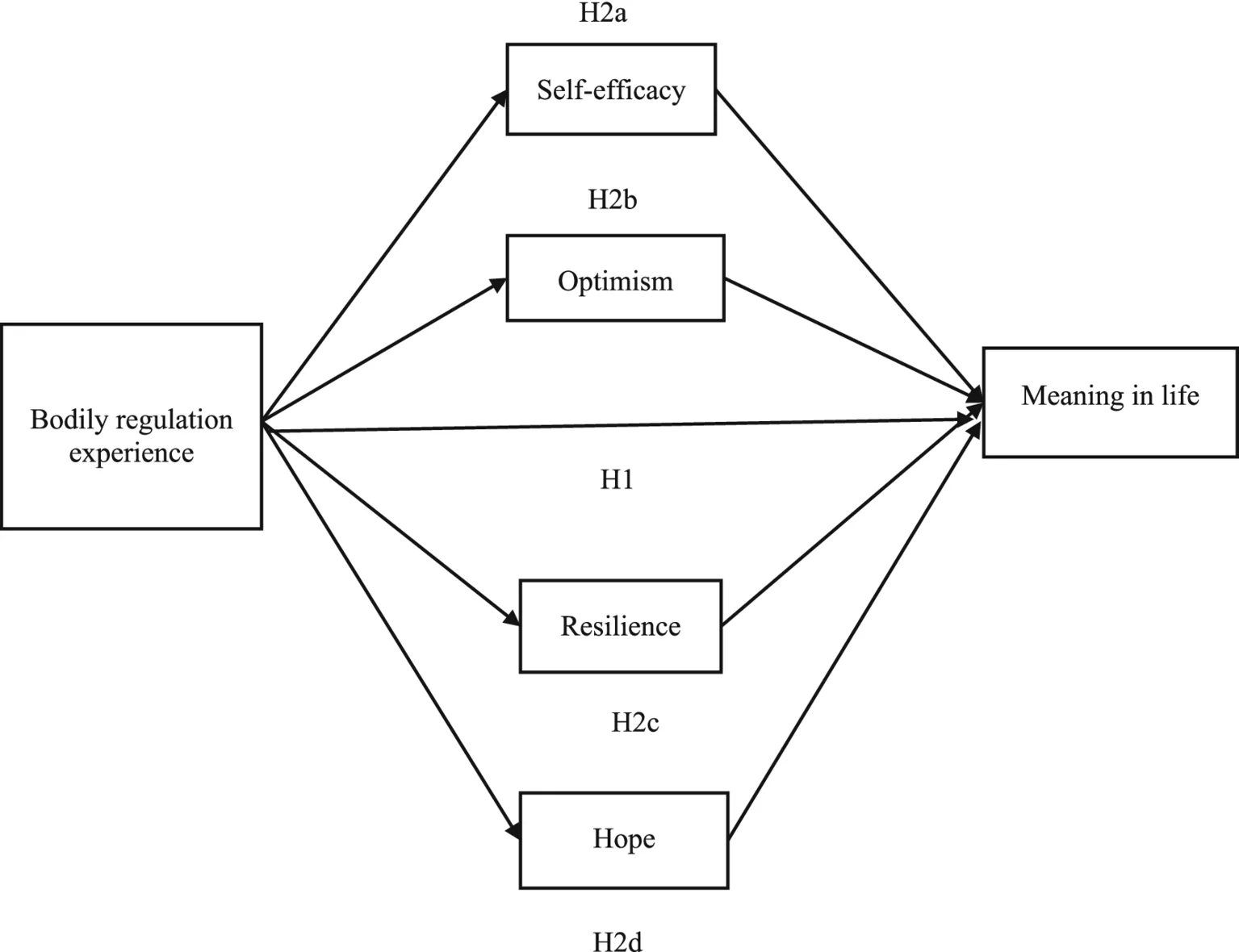
Research hypotheses model 1.

**Figure 2:**
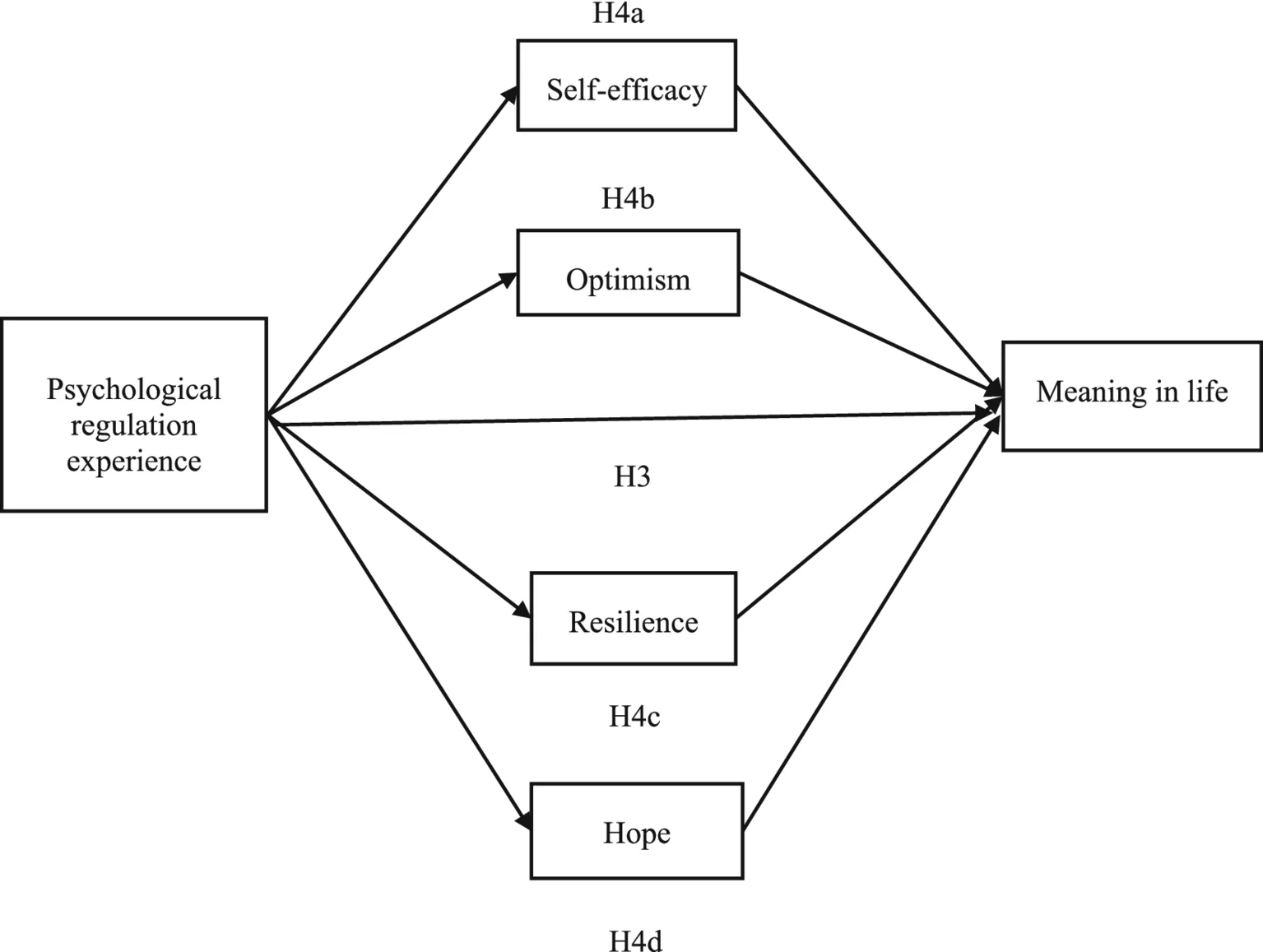
Research hypotheses model 2.

*H1*: Bodily regulation experience is positively associated with meaning in life among older adults.

*H2a*: Self-efficacy mediates the relationship between bodily regulation experience and older adults’ meaning in life.

*H2b*: Optimism mediates the relationship between bodily regulation experience and older adults’ meaning in life.

*H2c*: Resilience mediates the relationship between bodily regulation experience and older adults’ meaning in life.

*H2d*: Hope mediates the relationship between bodily regulation experience and older adults’ meaning in life.

*H3*: Psychological regulation experience is positively associated with meaning in life among older adults.

*H4a*: Self-efficacy mediates the relationship between psychological regulation experience and older adults’ meaning in life.

*H4b*: Optimism mediates the relationship between psychological regulation experience and older adults’ meaning in life.

*H4c*: Resilience mediates the relationship between psychological regulation experience and older adults’ meaning in life.

*H4d*: Hope mediates the relationship between psychological regulation experience and older adults’ meaning in life.

## 3 Study 1: scale development and validation

### 3.1 Methods

#### 3.1.1 Thematic coding of scale dimensions

This study used semi-structured interviews to conduct in-depth interviews with five experts (coded E1–E5) with extensive experience in Health Qigong instruction and research, and ten older adults (coded P1–P10) who practiced Health Qigong. The interview guide included questions such as “As a senior Health Qigong expert, where did you see its most evident benefits?” “What bodily and mental changes did you experience during practice?” and “Which experiences left the deepest impression on you?” These questions mainly encouraged participants to describe their embodied experience in natural language. After verbatim transcription of all interview records, the textual data were analyzed using thematic analysis. The analysis followed the procedures outlined by Braun and Clarke (54): first, repeated reading of the transcripts to familiarize oneself with the data; second, initial coding of statements related to Health Qigong embodied experience, yielding a total of 60 initial codes; third, grouping similar codes into six preliminary themes; fourth, through repeated comparison and review, integrating these preliminary themes; fifth, refining the integrated preliminary themes; and finally, extracting two core themes with clear definitions and labels. Tables 1, 2 present detailed procedures.

**Table 1:** Thematic analysis coding for bodily regulation experience.

Original statement (respondent code)	Initial codes	Preliminary theme	Core theme
“When practicing Baduanjin movements, I can feel the muscles in my arms and back being slowly stretched, and my breathing becomes deep and long along with the movements, making me feel qi moving inside my body.” (P1) “When practicing, I pay attention to keeping my spine straight, and I can feel a pulling sensation along a straight line from my head to my tailbone, as if my whole body is being elongated.” (P2) “After standing for a long time, I feel my legs are rooted to the ground like a tree, my toes gripping the floor, and my calf muscles twitching rhythmically—this is a sign of qi and blood circulation.” (P3)	Muscle stretching sensation; Deepened breathing; Spinal elongation sensation; Body uprightness; Toes gripping sensation; Rhythmic muscle twitching	Body perception	Bodily regulation experience
“Every time I practice Wuqinxi, my body sways left and right, I can feel the muscles around my waist and abdomen exerting force and after finishing, my entire back feels warm.” (P4) “During practice, each time I exhale, I can feel my lower abdomen bulging like a balloon being inflated, and after finishing, my abdomen remains warm.” (P5) “During practice, I feel that saliva secretion increases significantly, and after swallowing, my throat feels moist and smooth.” (P8)	Muscle exertion sensation; Body warmth; Lower abdomen bulging sensation; Abdominal warmth; Increased saliva secretion; Throat moistness		
“When breathing, I make different sounds with my mouth, and I can clearly feel my chest and abdominal cavities vibrating. My breathing has become much more even than before.” (P6) “When inhaling, I feel the airflow entering through my nose, traveling down my throat to my chest; when exhaling, it slowly flows out from my chest. My entire airway feels unobstructed.” (P7)	Chest cavity vibration; Abdominal breathing; Even breathing; Smooth airflow; Visceral traction; Enhanced cardiopulmonary function	Body regulation	
“From teaching observations, early practice feedback mostly focuses on the physical level, such as ‘my waist does not ache,’ ‘my legs feel strong,’ and ‘I sweat slightly.’” (E1) “Through experiments, we found that after practice, older adults’ proprioceptive thresholds significantly decrease, meaning they perceive their joint positions more accurately, thereby reducing fall risk.” (E2)	Joint comfort; Reduced body sway; Surface body warmth; Enhanced proprioception; Improved joint position sense; Increased balance ability	Somatosensory experience	
“After older adults practice Baduanjin, postural assessment shows improvement in spinal curvature, especially noticeable reduction in thoracic kyphosis.” (E3) “We compared older adults’ respiratory function indicators before and after practice and found significant increases in maximum ventilation volume and peak expiratory flow rate, indicating that respiratory muscle strength and endurance have been trained.” (E4)	Improvement of spinal curvature; Thoracic kyphosis is alleviated; Unobstructed breathing; Enhanced expiratory muscle strength; Improved ventilation function; Ontology feedback enhancement		

**Table 2:** Thematic analysis coding for psychological regulation experience.

Original statement (respondent code)	Initial codes	Preliminary theme	Core theme
“I used to be impatient and easily agitated. When practicing Liuzijue, I focus my attention on the liver area. As I exhale with sound, I feel the inner irritation subside and become particularly calm.” (P2) “When practicing Wuqinxi, I look backward and twist my body. At that moment, distracting thoughts disappear. There is only the present movement and breath, and I feel especially happy after practice.” (P3)	Attentional focus; Emotional soothing; Inner peace; Pleasant mood; Eliminating distractions; Clearing the mind	Psychological perception	Psychological regulation experience
“Sometimes I feel restless and do not want to talk. But after practice, following the movements and breath, I also expel mental distractions. I feel much more relaxed, can quiet myself down, and my mood improves.” (P9) “I have high blood pressure and get dizzy when anxious. After practicing Liuzijue, when facing something urgent, I can adjust my state through vocal exhalation and not panic.” (P10)	Letting go of distractions; Self-awareness; Calming the mind and spirit; Emotional regulation; Physiological feedback; Pleasant mood		
“I started practicing because my spouse passed away and I had insomnia. After 3 months of Baduanjin, my sleep improved greatly, and I felt much more at peace.” (P7) “I used to feel life was meaningless because my children are not often around. Since practicing Wuqinxi, I have met a group of old friends; we practice together, life has become meaningful, and I have something to look forward to.” (P8)	Reduced rumination; Improved sleep; Self-acceptance; Social belonging; Sense of meaning in life; Subjective well-being	Psychological regulation	
“From a cognitive psychology perspective, the attentional guidance training in Health Qigong is essentially a practice of cognitive reappraisal—actively shifting attention from negative thoughts to bodily sensations, forming a habitual emotion regulation strategy through repeated practice.” (E2) “In our focus group discussions, many older adults who had practiced for more than a year mentioned that they learned to ‘anchor with breath.’ Whenever their emotions fluctuated, they immediately returned to focusing on their breath. The automaticity of this emotion regulation increased, and their psychological state improved accordingly.” (E3)	Cognitive reappraisal; Negative thought shifting; Emotion regulation strategy; Breath anchoring; Automatic emotion regulation; Improved psychological state	Psychological restoration	
“A retired teacher is a typical case: She felt empty due to the loss of her social role. Later, she became a group leader in a Health Qigong team, leading collective practice. This regained sense of being needed reflects the reconstructive function of embodied practice on self-identity.” (E4) “We observed that the group practice format in Health Qigong naturally promotes social connection and emotional resonance among members through synchronized movements and breathing rhythms, which helps alleviate loneliness in older adults.” (E5)	Social role reshaping; Self-identity reconstruction; Self-worth recognition; Enhanced social connection; Increased emotional resonance; Reduced loneliness		

The statements in Table 1 focus on observable, controllable physiological aspects, including movement patterns, breathing rhythm, muscle tension, and thermal sensation. Corresponding initial codes include “muscle stretching sensation”, “deepened breathing”, “body warmth”, “abdominal breathing”, and “joint comfort.” These codes are grouped under the preliminary themes of “body perception, body regulation, and somatosensory experience”, which are then integrated into the core theme of “bodily regulation experience”.

The statements in Table 2 involve active attentional regulation, a transition from agitation to calm, and internal awareness and acceptance. Initial codes include “attentional focus”, “emotional soothing”, “inner peace”, “releasing distractions”, and “self-awareness.” These codes are grouped under the preliminary themes of “psychological perception, psychological regulation, and psychological restoration,” which are then integrated into the core theme of “psychological regulation experience”.

In summary, the accounts of experts and older adults showed high consistency. Respondents consistently reported two interrelated yet distinct types of experiences. The first pertained to observable, controllable physiological aspects, including movement patterns, breathing rhythm, muscle sensations, and body posture, as illustrated by the following: “The movements are slow, round, lively, and the breathing becomes deep and long”. “I can feel the qi (vital energy) moving inside my body”. The second involved changes and regulation of internal psychological states, including attentional focus and shifting, the transition from anxiety to calm, and the acceptance and awareness of one’s internal state, as reflected in the following: “I feel particularly happy after practice”. “I’m no longer so restless inside, and I can be quietly with myself for a while”. Our research team subsequently named these two types “bodily regulation experience” and “psychological regulation experience” respectively. The independent coding consistency coefficients (Kappa) exceeded 0.85, indicating high reliability of the categorization.

No new themes emerged during the later stages of coding, indicating that data saturation was reached. Tables 1, 2 present the complete process from initial coding to theme integration, providing an audit trail for the analysis. The interview sample included both experts and older adult practitioners, and the subsequent scale development and pilot testing were grounded in the interview findings, together constituting data source and method triangulation. The research team members had backgrounds in Health Qigong and sport psychology, and the team maintained reflective discussions throughout the analysis to minimize subjective bias. Formal member checking was not conducted in this study, for two main reasons. First, the older adult participants were geographically dispersed, which limited follow-up access. Second, the core themes, distilled through multiple rounds of coding into abstract academic constructs, exceeded the everyday expressive range of the respondents; accordingly, direct follow-up verification might have introduced comprehension bias. This limitation warrants attention in future research.

#### 3.1.2 Formation of the preliminary scale

Although Health Qigong has been widely studied, most research has treated it primarily as an intervention tool, focusing on its outcomes rather than on practitioners’ embodied experience. As a result, no scale has yet been developed to measure the embodied experience of Health Qigong. Most studies have treated Health Qigong merely as an intervention tool. In this study, we designed scale items grounded in the health-preserving features of Health Qigong, “body regulation, breath regulation, and mind regulation,” in conjunction with the embodied cognition principles of “immersiveness, situatedness, and interactivity.” We first collected and compiled initial items by reviewing relevant books and literature, as well as older adults’ embodied accounts of daily Health Qigong practice. Items were constructed from two dimensions: bodily regulation experience and psychological regulation experience (9). The scale aimed to capture older adults’ refined perceptions and experiences of their physical state, movement processes, and breath flow arising from the coordination of limb movements and breathing. Additionally, it aimed to explore how older adults, through the coordination of movement and breathing, achieve a personally experienced state of mental tranquility through attentional control, focused concentration, and emotional regulation.

Subsequently, the research team developed and analyzed the scale items, resulting in 30 items. We invited five experts from Guangzhou Sport University to independently evaluate the validity of each item. After discussion, the research team selected 12 items with high validity ratings from the item pool for each factor, forming the preliminary “Health Qigong Bodily Regulation Experience” and “Health Qigong Psychological Regulation Experience” scales. All items were rated on a five-point Likert scale, with participants selecting responses ranging from “1 = strongly disagree” to “5 = strongly agree” based on their actual situation. All items were positively worded, and higher scores indicated stronger levels of bodily and psychological regulation experience in Health Qigong among older adults.

#### 3.1.3 Preliminary samples and testing procedures

In December 2025, questionnaires were distributed to a convenience sample of 150 older adults across six communities in Guangzhou, yielding 120 valid responses (49 men, 71 women). The inclusion criteria were as follows: aged 60 to 75 years, regular Health Qigong practice experience, no diagnosed cognitive impairment or psychiatric disorder, and no severe visual or auditory impairment that would preclude normal communication. During the preliminary testing phase, only the self-developed 12-item Health Qigong Embodied Experience Scale was administered.

The collected data were subjected to reliability analysis and exploratory factor analysis (EFA). Item discrimination was assessed using the corrected item-total correlation (CITC), defined as the Pearson correlation between each item and the total score of the remaining items, with a threshold of 0.5. The second item of the Health Qigong Bodily Regulation Experience and the third item of the Health Qigong Psychological Regulation Experience yielded CITC values below 0.5 and, in the EFA, failed to meet the unidimensionality criterion; these two items were therefore removed. The resulting preliminary scale comprised two factors and 10 items.

## 4 Results

### 4.1 Reliability testing of the preliminary scale

In this study, SPSS 27.0 was used to test the reliability of all items in the Health Qigong Bodily Regulation Experience and Health Qigong Psychological Regulation Experience scales. The results showed that the Cronbach’s α values for the preliminary bodily regulation experience and psychological regulation experience scales were 0.893 and 0.807, respectively, both exceeding 0.8 and indicating strong internal consistency. As shown in Table 3, for both preliminary scales, the α coefficients after deleting any item were lower than the original α coefficients. The reliability indices reached optimal levels, and the corrected item-total correlation (CITC) values for all items exceeded 0.5, with higher values indicating greater internal consistency between each item and the remaining items. Therefore, all measurement items in the preliminary scales were retained.

**Table 3:** Cronbach’s reliability analysis results for the two scales.

Total items	Corrected item-total correlation	Cronbach’s alpha if item deleted	Cronbach’s alpha
BRE 1	0.735	0.872	0.893
BRE 2	0.786	0.858	
BRE 3	0.727	0.872	
BRE 4	0.708	0.876	
BRE 5	0.742	0.868	
PRE 1	0.647	0.752	
PRE 2	0.578	0.774	
PRE 3	0.532	0.787	0.807
PRE 4	0.630	0.757	
PRE 5	0.574	0.775	

BRE, Bodily Regulation Experience, PRE, Psychological Regulation Experience.

### 4.2 Validity testing of the preliminary scale

In this study, SPSS 27.0 was used to test the validity of all items in the Health Qigong Bodily Regulation Experience and Health Qigong Psychological Regulation Experience scales. The KMO value was 0.848 (> 0.8), indicating good validity. Bartlett’s test of sphericity yielded 561.866 (*p* < 0.001), rejecting the null hypothesis and confirming the presence of common factors among the variables; thus, the data were suitable for factor analysis. Principal component analysis was employed for factor extraction using varimax orthogonal rotation. During extraction, only factors with eigenvalues greater than 1 were retained. Two principal factors were extracted, explaining 35.141 and 63.521% of the variance, respectively. The results are shown in Table 4.

**Table 4:** Total variance explained.

Component	Initial eigenvalues			Extraction sums of squared loadings			Rotation sums of squared loadings		
	Total	% of variance	Cumulative %	Total	% of variance	Cumulative %	Total	% of variance	Cumulative %
1	4.721	47.211	47.211	4.721	47.211	47.211	3.514	35.141	35.141
2	1.631	16.309	63.521	1.631	16.309	65.521	2.838	28.38	65.521

Table 5 presents the rotated factor loadings. As shown, each factor contained five items, and all item loading coefficients exceeded 0.5. These factors were consistent with the originally constructed dimensions, and no item cross-loaded onto another factor. The first common factor was named “Bodily Regulation Experience”, and the second was named “Psychological Regulation Experience”. Thus, the reliability and validity of the preliminary scale were established. Formal measurement using the two scales and confirmatory factor analysis (CFA) are presented in the section that follows.

**Table 5:** Rotated factor loading matrix results.

Variables	Items	Factor loading coefficient	
		Factor 1	Factor 2
Bodily regulation experience	BRE 1	0.811	
	BRE 2	0.840	
	BRE 3	0.828	
	BRE 4	0.773	
	BRE 5	0.813	
Psychological regulation experience	PRE 1		0.760
	PRE 2		0.725
	PRE 3		0.743
	PRE 4		0.632
	PRE 5		0.760

BRE, Bodily Regulation Experience; PRE, Psychological Regulation Experience.

## 5 Study 2: formal survey and scale validation

### 5.1 Participants

To promote the preservation and development of Health Qigong and to gain insight into older adults’ authentic physical and psychological experiences following Health Qigong practice, as well as its role in fostering meaning in life, the research team first conducted field visits to parks, squares, and other exercise venues across six communities in Guangzhou between December 2025 and January 2026. The visits examined the overall profile of Health Qigong practice among older adults in Guangzhou in terms of practice content, duration, frequency, and perceived effects, while also yielding in-depth insights into the key physical and psychological changes older adults experienced after practicing Health Qigong and their understanding of psychological capital and meaning in life.

Second, this study adopted a convenience sampling method to survey 530 older adults across six communities in Guangzhou. The six communities were selected to cover different administrative districts of Guangzhou, thereby capturing older adults from diverse socioeconomic backgrounds. Inclusion criteria were as follows: (1) Age: 60–75 years. This age range is classified by the World Health Organization as the young-old, a population characterized by a relatively gradual and optimistic trajectory of physical and psychological decline, with the capacity for independent living. (2) Health status: no cognitive impairment or mental illness, no severe visual or hearing impairment, and the ability to communicate normally. (3) Experience in Health igong practice, either past or current. All participants took part voluntarily. Exclusion criteria were failure to meet any of the above criteria or submission of an invalid questionnaire.

Finally, with the assistance of community administrators, electronic questionnaire links were distributed to older adults in the communities through two channels: online announcements posted in community WeChat groups and on-site QR code scanning at community events. Questionnaires were distributed and collected between March 5 and March 10, 2026. However, given the non-probability nature of convenience sampling, this sample represents older adult Health Qigong practitioners in Guangzhou communities, and the findings are limited to this population.

### 5.2 Procedure

First, this study adhered to the ethical principles of the Declaration of Helsinki and received approval from the Ethics Committee of Guangdong University of Science and Technology. Second, participants were informed of the research purpose, which was solely scientific investigation, and the voluntary nature of participation was emphasized; they were free to withdraw at any time. Third, respondents were asked about their engagement in daily Health Qigong practice and their familiarity with and evaluation of Health Qigong. They then answered questions on psychological capital and meaning in life based on their recent physical and mental states, and provided basic demographic information. Fourth, we invited participants to sign the consent form. Finally, community administrators in Guangzhou strongly supported this study and helped distribute the questionnaire online. All participants completed the questionnaire anonymously, with assistance from their family members.

This study controlled for seven demographic variables, including gender and age, when examining the associations between Health Qigong embodied experience and older adults’ meaning in life, as well as the mediating role of psychological capital. After collecting the questionnaires, our research team conducted rigorous data checking and management, reviewing the response quality of each questionnaire individually. Responses that were randomly completed or in which the same option was consistently selected throughout were considered invalid and excluded. We excluded such responses because they reflected a lack of comprehension of questionnaire items and a casual or perfunctory approach to participation. More importantly, these invalid data could not accurately capture the relationships among Health Qigong embodied experience, meaning in life, and psychological capital. Including them would have compromised the accuracy of the overall association and mediation analyses, undermining the credibility of the research conclusions.

After screening, a total of 530 questionnaires were distributed to older adults. Of these, 500 valid questionnaires were returned, yielding an effective response rate of 94%. The survey included 500 older adults: 237 men and 263 women. As for marital status, 479 were married, 7 were unmarried, 4 were divorced, and 10 were widowed. Regarding children, 289 had multiple children, 208 had only one child, and 3 had no children. Regarding the frequency of Health Qigong practice, 446 practiced frequently, 30 practiced occasionally, and 24 practiced rarely.

### 5.3 Measures

#### 5.3.1 Health Qigong Embodied Experience Scale

In the preliminary study, the Health Qigong Bodily Regulation Experience Scale and the Psychological Regulation Experience Scale were designed and developed, and the reliability and validity of both scales were confirmed. During the formal investigation, reliability testing was repeated for both scales. Cronbach’s α coefficients showed that the α value was 0.916 for bodily regulation experience and 0.922 for psychological regulation experience, further confirming that both scales had good reliability.

#### 5.3.2 Psychological Capital Scale

Drawing on the Psychological Capital Questionnaire (PCQ) developed by Luthans et al. (33), this study adapted a Psychological Capital Scale tailored to the research context by integrating the characteristics of older adults’ embodied Health Qigong practice. We adopted a published standardized Chinese translation of the PCQ (34), with item wording contextually adapted to the Health Qigong practice setting, and no independent translation and back-translation procedure was conducted. The research team developed the initial draft of the adapted items through collective discussion and then reviewed them by two sport psychology experts from Guangzhou Sport University, who evaluated each adapted item to ensure it remained faithful to the core meaning of the original construct after contextualization. Guided by three principles: theoretical fidelity, contextualized adaptation, and participant-friendliness, the items were revised to measure the four dimensions. Illustrative examples include “If I keep practicing Health Qigong, my physical and mental state will continue to improve” (optimism), “When learning complex or difficult qigong routines, I have the patience and determination to master them” (resilience), “Regarding Health Qigong practice, I have clear short-term goals and long-term expectations” (hope), and “I am confident that I can master the movement principles and breathing techniques taught by the instructor” (self-efficacy).

The scale comprises four dimensions, each containing five items, yielding a total of 20 items. All items were rated on a five-point Likert scale, with participants selecting responses from “1 = strongly disagree” to “5 = strongly agree” based on their actual situation. All items were positively worded, with higher scores reflecting higher overall levels of psychological capital. Cronbach’s α coefficients for the four dimensions were 0.936 (optimism), 0.841 (resilience), 0.876 (hope), and 0.911 (self-efficacy), indicating good reliability for each subscale. In the confirmatory factor analysis, the composite reliability (CR) and average variance extracted (AVE) of the four dimensions all met the recommended thresholds (see Table 6), and discriminant validity among the dimensions was satisfactory (see Table 7), confirming that the adapted scale retained good construct validity.

**Table 6:** Questionnaire items and CFA results.

Construct	Dimension	Item	Loading	T-value	CR	AVE
Health Qigong embodied experience	Bodily regulation experience	1. In the rise and fall, opening and closing of the movements, my breathing rhythm can naturally adjust accordingly, forming a coherent internal rhythm.	0.877	-	0.919	0.693
		2. When maintaining a certain posture, I can keep my body relaxed and stable through steady breathing.	0.801	22.966		
		3. I can perceive through physical exercises how breathing drives the circulation and flow of the internal qi within my body.	0.857	25.937		
		4. Every inhalation and exhalation makes me feel more clearly the close connection between my life activities and the current action situation.	0.779	21.889		
		5. After practicing for a period of time, the interaction between movements and breathing can promote the smooth flow of qi and blood in my body and enhance my physical functions.	0.844	25.19		
	Psychological regulation experience	6. When practicing, my attention can naturally focus on the current movement and physical sensations, and it's not easy for me to get distracted.	0.848	-	0.923	0.705
		7. The slow rhythm of the movements can guide my mind to gradually calm down and eliminate unnecessary distractions.	0.839	23.609		
		8. The sense of relief and pleasure gained after practice is a positive experience of life that naturally emerges through the process of physical activity.	0.784	21.184		
		9. In the natural environment of practicing martial arts, my consciousness is immersed in it, and my mind reaches a state of harmony and unity with the outside world.	0.852	24.253		
		10. The “relaxation” felt by my body and the “calmness” perceived by my spirit are an experience of the unity of body and mind.	0.872	25.207		
Psychological capital	Optimism	1. I believe that by persistently practicing health Qigong, my physical and mental condition will become better and better.	0.879	-	0.937	0.747
		2. Even when I feel unwell, I believe that health Qigong can help me recover quickly.	0.915	30.231		
		3. When practicing Qigong, I am confident that each practice can bring some positive feelings.	0.85	25.955		
		4. Overall, I am optimistic about improving the quality of life through continuous practice of Qigong.	0.84	25.385		
		5. I tend to think that any minor difficulty encountered in qigong practice can eventually be overcome.	0.837	25.229		
	Resilience	6. If my practice is interrupted for a few days due to special reasons (such as visiting relatives and friends), I can quickly get back on track and keep up with the pace.	0.815	-	0.847	0.529
		7. When learning complex or difficult Qigong routines, I have the patience and determination to master and practice them well.	0.765	18.483		
		8. Even if my body feels a little sore and swollen after practice, I can adjust and keep going without giving up easily.	0.765	18.482		
		9. When I encounter troubles in life, I can help myself regain calm by focusing on qigong practice.	0.581	13.239		
		10. Facing some physical changes brought about by aging, I think that through regular practice, I can adapt well.	0.688	16.178		
	Hope	11. For the practice of health Qigong, I have clear short-term goals (such as mastering a certain movement this month) and long-term expectations.	0.798	-	0.879	0.593
		12. When the practice progresses slowly, I can come up with different methods (such as breaking down the movements and increasing the practice frequency) to break through.	0.821	20.237		
		13. I am convinced that as long as I use the right methods and persevere, I can achieve the health goals I expect through Qigong.	0.792	19.317		
		14. In the pursuit of health, I think practicing Qigong is a clear and effective way.	0.678	15.935		
		15. I am gradually approaching the physical and mental health goals I have set for myself through planned practice.	0.755	18.18		
	Self-efficacy	16. I am confident that I can master the key points of the movements and breathing methods of the health qigong taught by the teacher.	0.822	-	0.911	0.672
		17. I believe I can persist in practicing health Qigong regularly for a long time.	0.815	21.198		
		18. In group practice, I can coordinate myself well without affecting the whole, and also gain strength from the collective atmosphere.	0.789	20.242		
		19. I believe I can effectively relieve daily stress and fatigue by practicing health Qigong.	0.817	21.288		
		20. Overall, I think I'm doing better and better in learning and practicing health Qigong.	0.855	22.743		
Meaning in life	Presence of meaning	1. I can always manage to solve difficult problems if I try hard enough.	0.657	-	0.903	0.511
		2. If someone opposes me, I can find the means and ways to get what I want.	0.782	15.267		
		3. I am certain that I can accomplish my goals.	0.598	12.125		
		4. I am confident that I could deal efficiently with unexpected events.	0.762	14.943		
		5. Thanks to my resourcefulness, I can handle unforeseen situations.	0.619	12.509		
	Search of meaning	6. I can solve most problems if I invest the necessary effort.	0.663	13.285		
		7. I can remain calm when facing difficulties because I can rely on my coping abilities.	0.78	15.233		
		8. When I am confronted with a problem, I can find several solutions.	0.749	14.731		
		9. If I am in trouble, I can think of a good solution.	0.789	15.388		

CR, Composite reliability; AVE, Average variance extracted.

**Table 7:** Discriminant validity of measures and correlation matrix.

	BRE	PRE	Optimism	Resilience	Hope	Self-efficacy	Meaning in life
BRE	**0.833**						
PRE	0.631	**0.839**					
Optimism	0.569	0.583	**0.865**				
Resilience	0.628	0.687	0.555	**0.727**			
Hope	0.668	0.678	0.675	0.617	**0.770**		
Self-efficacy	0.721	0.583	0.507	0.542	0.615	**0.820**	
Meaning in life	0.636	0.686	0.568	0.703	0.638	0.538	**0.715**

The numbers on the diagonal row are the square root of Average Variance Extracted (AVE). Off-diagonal values are the correlations between the constructs. BRE, Bodily Regulation Experience, PRE, Psychological Regulation Experience.

#### 5.3.3 The Meaning in Life Scale

Drawing on the Meaning in Life Questionnaire (MLQ) developed by Steger et al. (11), this study adopted the Chinese version validated by Liu and Gan (35). Because the MLQ items consist of general statements about meaning in life, unconstrained by specific occupations or behavioral contexts, they apply equally well to older adult Health Qigong practitioners; accordingly, no modifications were made to the item wording. The original scale comprises two dimensions, presence of meaning and search for meaning, each containing five items, for a total of 10 items. During measurement, one item in the search for meaning dimension had a low factor loading and was removed, resulting in a final set of nine items: five items for presence of meaning and four for search for meaning.

All items were rated on a five-point Likert scale, with participants selecting responses from “1 = strongly disagree” to “5 = strongly agree” based on their actual situation. All items were positively worded, with higher scores indicating a stronger sense of meaning in life. In the present sample, the Cronbach’s α coefficient was 0.815 for the presence of meaning subscale (M = 4.14, SD = 0.622) and 0.813 for the search for meaning subscale (M = 4.55, SD = 0.652), indicating acceptable internal consistency for both dimensions. The two subscales were significantly and moderately correlated (r = 0.548, *p* < 0.001), suggesting that presence of meaning and search for meaning are not independent constructs. The Cronbach’s α coefficient for the full nine-item scale was 0.903, indicating good overall reliability. In the confirmatory factor analysis, the composite reliability (CR) and average variance extracted (AVE) met the recommended thresholds (see Table 6), and discriminant validity between the two dimensions was satisfactory (see Table 7), confirming that the scale retained good construct validity. Given that this study focused on the overall sense of meaning in life rather than the differential effects of presence versus search, the MLQ total score was used in all subsequent analyses.

### 5.4 Data analysis

This study used AMOS 26.0 to conduct CFA to evaluate the composite reliability (CR) and average variance extracted (AVE) of the measurement model. The factor loadings ranged from 0.581 to 0.915 (see Table 6). The CR values for bodily regulation experience (0.919), psychological regulation experience (0.923), optimism (0.937), resilience (0.847), hope (0.879), self-efficacy (0.911), and meaning in life (0.903) all exceeded the 0.70 benchmark, further supporting internal consistency and reliability. The AVE values for bodily regulation experience (0.693), psychological regulation experience (0.705), optimism (0.747), resilience (0.529), hope (0.593), self-efficacy (0.672), and meaning in life (0.511) all exceeded the 0.50 benchmark, indicating high convergent validity. The overall fit indices of the model were χ^2^/df = 2.704, CFI = 0.921, NFI = 0.881, IFI = 0.922, TLI = 0.915, RMSEA = 0.058 (90% CI = [0.055, 0.062]), and SRMR = 0.042, indicating a good fit between the measurement model and the data. Furthermore, discriminant validity was confirmed through the correlation matrix. The matrix showed that the square root of the AVE for each construct was greater than the correlation coefficients between constructs, indicating strong discriminant validity (see Table 7). Overall, the measurement model demonstrated good reliability and validity, providing a solid foundation for testing the research hypotheses.

Additionally, SPSS 27.0 was first used to conduct a common method bias test on the questionnaire data to ensure the data were free from bias. Next, descriptive statistics and correlation analysis were performed to describe the basic characteristics of the variables and to examine the linear relationships among them. Finally, Model 4 of the PROCESS 4.0 macro was used to analyze the mediating pathway and to examine the associations between Health Qigong embodied experience and older adults’ meaning in life. The mediating roles of the dimensions of psychological capital in the relationships between Health Qigong bodily regulation experience, psychological regulation experience, and meaning in life were also explored. In the mediation analysis, the bootstrapping method (5,000 resamples) was used to construct confidence intervals for the mediating pathway of the core variables, to produce more robust results.

## 6 Results of study 2

### 6.1 Common method bias test

Since all the data in this study were self-reported by older adults, Harman’s single-factor test was conducted to examine common method bias. The results showed eight factors with eigenvalues greater than 1. The first factor accounted for 13.20% of the total variance, which was below the 40% threshold, indicating that common method bias was not a serious concern in this study.

### 6.2 Descriptive statistics and correlation analysis

Descriptive statistics and correlation analysis were performed on the seven variables, with the results presented in Table 8. All pairwise correlations among bodily regulation experience, psychological regulation experience, optimism, resilience, hope, self-efficacy, and meaning in life were significantly positive (*p* < 0.01). Accordingly, the conditions were met for subsequent analyses of the total association and mediating pathway examined in this study.

**Table 8:** Descriptive statistics and correlation analysis for the variables (*N* = 500).

Variables	BRE	PRE	Optimism	Resilience	Hope	Self-efficacy	Meaning in life
BRE	1						
PRE	0.631^**^	1					
Optimism	0.529^**^	0.553^**^	1				
Resilience	0.594^**^	0.662^**^	0.537^**^	1			
Hope	0.617^**^	0.635^**^	0.656^**^	0.606^**^	1		
Self-efficacy	0.691^**^	0.558^**^	0.490^**^	0.535^**^	0.614^**^	1	
Meaning in life	0.593^**^	0.651^**^	0.556^**^	0.694^**^	0.623^**^	0.532^**^	1
M	4.43	4.52	4.18	4.43	4.32	4.20	4.29
SD	0.577	0.580	0.568	0.498	0.548	0.754	0.511

Significance level: ^**^p<0.01. BRE, Bodily Regulation Experience, PRE, Psychological Regulation Experience.

### 6.3 Test for the mediating role

This study included gender, age, income, marital status, number of children, physical condition, and practice frequency as control variables. The SPSS PROCESS macro (Model 4) was used with 5,000 bootstrap resamples and 95% bias-corrected confidence intervals to examine the mediating roles of optimism, resilience, hope, and self-efficacy in the associations of bodily regulation experience and psychological regulation experience with meaning in life. A mediating role was considered significant when the confidence interval did not include zero. Detailed results of the mediation analyses are presented in the following tables.

#### 6.3.1 Total association

The results (see Table 9) showed that after controlling for all demographic variables, gender (*β* = −0.013, t = −0.35), age (*β* = −0.053, t = −1.46), income (*β* = −0.019, t = −0.485), marital status (*β* = −0.092, t = −2.496), number of children (*β* = −0.011, t = −0.285), and physical condition (*β* = 0.036, t = 0.915) were not significantly associated with meaning in life. By contrast, practice frequency (*β* = 0.087, t = 2.07) was significantly and positively associated with meaning in life. The overall association remained unchanged after including covariates. Health Qigong bodily regulation experience was significantly associated with older adults’ meaning in life (*β* = 0.532, t = 13.017, *p* < 0.01). The overall model was statistically significant, *F* (8, 491) = 36.403, *p* < 0.001, R^2^ = 0.372, adjusted R^2^ = 0.362. All tolerance values exceeded 0.1, and the variance inflation factor (VIF) values ranged from 1.030 to 1.872, all below the threshold of 2, indicating that multicollinearity was not a concern. These findings aligned with the hypothesized association between Health Qigong bodily regulation experience and older adults’ meaning in life, supporting Hypothesis H1.

**Table 9:** Mediating pathway of Health Qigong bodily regulation experience on meaning in life (*N* = 500).

Variables	Meaning in life (total association)			Optimism			Resilience			Hope			Self-efficacy			Meaning in life (direct association)		
	*β*	SE	T	*β*	SE	T	*β*	SE	T	*β*	SE	T	*β*	SE	T	*β*	SE	T
Gender	−0.013	0.037	−0.35	−0.063	0.043	−1.664	0.047	0.036	1.292	0.023	0.038	0.676	0.044	0.049	1.359	−0.03	0.031	−1.011
Age	−0.053	0.036	−1.46	−0.016	0.042	−0.409	−0.06	0.035	−1.635	0.032	0.037	0.919	0.005	0.048	0.147	−0.033	0.03	−1.077
Income	−0.019	0.023	−0.485	−0.058	0.027	−1.427	−0.017	0.023	−0.43	−0.041	0.024	−1.116	0.027	0.031	0.776	0.001	0.019	0.022
Marital status	−0.092	0.059	−2.496^*^	−0.035	0.069	−0.915	−0.074	0.058	−2.011^*^	−0.09	0.06	−2.57*	0.014	0.079	0.43	−0.043	0.049	−1.394
Number of children	−0.011	0.03	−0.285	−0.021	0.035	−0.537	−0.024	0.029	−0.64	−0.037	0.031	−1.054	0.013	0.04	0.388	0.007	0.025	0.242
Physical condition	0.036	0.039	0.915	−0.064	0.046	−1.565	−0.005	0.039	−0.13	0.002	0.04	0.056	0.035	0.052	0.988	0.044	0.033	1.348
Practice frequency	0.087	0.024	2.07^*^	0.204	0.028	4.646**	0.021	0.024	0.506	0.243	0.025	6.065**	0.075	0.032	1.964	0.01	0.021	0.267
BRE	0.532	0.036	13.017^**^	0.453	0.042	10.62**	0.582	0.035	14.143^**^	0.504	0.037	12.991^**^	0.651	0.048	17.626^**^	0.126	0.041	2.708^**^
Optimism																0.119	0.037	2.889^**^
Resilience																0.409	0.042	10.01^**^
Hope																0.17	0.044	3.593^**^
Self-efficacy																0.044	0.03	1.013
*R^2^*	0.372			0.317			0.365			0.434			0.488			0.579		
Adjusted R^2^	0.362			0.306			0.355			0.425			0.48			0.569		
F	*F* (8,491) = 36.403, *p* = 0.000			F (8,491) = 28.440, *p* = 0.000			F (8,491) = 35.277, *p* = 0.000			F (8,491) = 47.074, *p* = 0.000			F (8,491) = 58.546, *p* = 0.000			*F* (12,487) = 55.854, *p* = 0.000		

Significance level: ^*^p < 0.05, ^**^p < 0.01, t-values in parentheses. BRE, Bodily Regulation Experience.

The results (see Table 10) showed that after controlling for all demographic variables, gender (*β* = −0.03, t = −0.881), age (*β* = −0.035, t = −1.006), income (*β* = 0.004, t = 0.114), marital status (*β* = −0.062, t = −1.796), number of children (*β* = 0.015, t = 0.442), and physical condition (*β* = 0.065, t = 1.787) were not significantly associated with meaning in life. By contrast, practice frequency (*β* = 0.082, t = 2.109) was significantly and positively associated with meaning in life. The overall association remained unchanged after including covariates. Health Qigong psychological regulation experience was significantly associated with older adults’ meaning in life (*β* = 0.593, t = 15.973, *p* < 0.001). The overall model was statistically significant, *F* (8, 491) = 49.087, *p* < 0.001, R^2^ = 0.444, adjusted R^2^ = 0.435. All tolerance values exceeded 0.1, and the variance inflation factor (VIF) values ranged from 1.030 to 1.872, all below the threshold of 2, indicating that multicollinearity was not a concern. These findings aligned with the hypothesized association between Health Qigong psychological regulation experience and older adults’ meaning in life, supporting Hypothesis H3.

**Table 10:** Mediating pathway of Health Qigong psychological regulation experience on meaning in life (*N* = 500).

Variables	Meaning in life (total association)			Optimism			Resilience			Hope			Self-efficacy			Meaning in life (direct association)		
	*β*	SE	T	*β*	SE	T	*β*	SE	T	*β*	SE	T	*β*	SE	T	*β*	SE	T
Gender	−0.03	0.035	−0.881	−0.078	0.042	−2.122	−0.028	0.034	0.824	0.006	0.036	0.179	0.017	0.055	0.474	−0.034	0.03	−1.145
Age	−0.035	0.034	−1.006	−0.001	0.041	−0.012	−0.04	0.033	−1.157	0.049	0.036	1.44	0.02	0.054	0.548	−0.028	0.03	−0.954
Income	0.004	0.022	0.114	−0.039	0.027	−0.992	0.008	0.022	0.221	−0.021	0.023	−0.58	0.045	0.035	1.16	0.005	0.019	0.17
Marital status	−0.062	0.056	−1.796	−0.013	0.067	−0.358	−0.043	0.054	−1.237	−0.066	0.059	−1.947	0.02	0.089	0.543	−0.037	0.048	−1.238
Number of children	0.015	0.028	0.442	−0.001	0.034	−0.017	0.004	0.028	0.118	−0.015	0.03	−0.442	0.027	0.045	0.709	0.014	0.024	0.479
Physical condition	0.065	0.037	1.787	−0.036	0.044	−0.921	0.028	0.036	0.755	0.033	0.039	0.927	0.092	0.058	2.348^*^	0.048	0.032	1.524
Practice frequency	0.082	0.023	2.109^*^	0.21	0.027	4.956*	0.018	0.022	0.467	0.251	0.024	6.555**	0.155	0.036	3.679^**^	0.008	0.02	0.214
PRE	0.593	0.033	15.973^**^	0.467	0.039	11.846^**^	0.642	0.032	17.301^**^	0.526	0.034	14.443^**^	0.481	0.052	12.01^**^	0.204	0.039	4.664^**^
Optimism																0.106	0.037	2.606^**^
Resilience																0.362	0.043	8.574^**^
Hope																0.143	0.044	3.023^**^
Self-efficacy																0.066	0.027	1.664
*R^2^*	0.444			0.346			0.445			0.466			0.354			0.591		
Adjusted R^2^	0.435			0.336			0.436			0.458			0.344			0.581		
F	F (8,491) = 49.087, *p* = 0.000			F (8,491) = 32.539, *p* = 0.000			F (8,491) = 49.166, *p* = 0.000			F (8,491) = 53.620, *p* = 0.000			F (8,491) = 33.646, *p* = 0.000			F (12,487) = 58.669, *p* = 0.000		

Significance level: ^*^p < 0.05, ^**^p < 0.01, t-values in parentheses. PRE, Psychological Regulation Experience.

#### 6.3.2 Mediating pathway of health Qigong bodily regulation experience on meaning in life

Table 9 shows that, after controlling for all demographic variables, the direct association between bodily regulation experience and meaning in life was statistically significant (*β* = 0.126, t = 2.708, *p* < 0.01). Regarding the indirect pathways, bodily regulation experience was significantly and positively associated with optimism (*β* = 0.453, t = 10.62, *p* < 0.01), and optimism was significantly and positively associated with meaning in life (*β* = 0.119, t = 2.889, *p* < 0.01). Following the same pattern, bodily regulation experience was significantly and positively associated with resilience (*β* = 0.582, t = 14.143, *p* < 0.01), and resilience was significantly and positively associated with meaning in life (*β* = 0.409, t = 10.01, *p* < 0.01). Likewise, bodily regulation experience was significantly and positively associated with hope (*β* = 0.504, t = 12.991, *p* < 0.01), and hope was significantly and positively associated with meaning in life (*β* = 0.170, t = 3.593, *p* < 0.01). Bodily regulation experience was also significantly and positively associated with self-efficacy (*β* = 0.651, t = 17.626, *p* < 0.01). However, the association between self-efficacy and meaning in life was not statistically significant (*β* = 0.044, t = 1.013, *p* > 0.05); the mediating role of self-efficacy was further examined through the bootstrap 95% confidence interval.

The results presented in Table 11 show that, for the model including self-efficacy, the indirect pathway value was 0.025 (*p* = 0.469 > 0.05), with a bootstrap 95% CI of [−0.046, 0.091]. The confidence interval included zero; the indirect pathway was not statistically significant, and no mediating role of self-efficacy was observed between bodily regulation experience and meaning in life. Hypothesis H2a was not supported. For the model including optimism, the indirect pathway value was 0.048 (*p* = 0.012 < 0.05), with a bootstrap 95% CI of [0.012, 0.086]. The confidence interval excluded zero; the indirect pathway was statistically significant. A partially mediating role of optimism between bodily regulation experience and meaning in life was observed, supporting Hypothesis H2b. For the model including resilience, the indirect pathway value was 0.211 (*p* < 0.001), with a bootstrap 95% CI of [0.138, 0.282]. The confidence interval excluded zero; the indirect pathway was statistically significant. A partially mediating role of resilience between bodily regulation experience and meaning in life was observed, supporting Hypothesis H2c. For the model including hope, the indirect pathway value was 0.076 (*p* = 0.003 < 0.01), with a bootstrap 95% CI of [0.028, 0.130]. The confidence interval excluded zero; the indirect pathway was statistically significant. A partially mediating role of hope between bodily regulation experience and meaning in life was observed, supporting Hypothesis H2d.

**Table 11:** Bootstrap analysis of mediation pathway.

Path	Value	Boot SE	Bootstrap 95% CI		*p*-value	Results
			Boot LLCI	Boot ULCI		
BRE → Optimism→ ML	0.048^*^	0.019	0.012	0.086	0.012	Support
BRE → Resilience→ ML	0.211^***^	0.037	0.138	0.282	0.000	Support
BRE → Hope→ ML	0.076^**^	0.026	0.028	0.130	0.003	Support
BRE → SE → ML	0.025	0.035	−0.046	0.091	0.469	Not support
PRE → Optimism→ ML	0.045^*^	0.019	0.009	0.082	0.017	Support
PRE → Resilience→ ML	0.205^***^	0.034	0.139	0.269	0.000	Support
PRE → Hope→ ML	0.066^**^	0.025	0.018	0.115	0.008	Support
PRE → SE → ML	0.028	0.025	−0.024	0.076	0.271	Not support

Significance level: ^*^p < 0.05, ^**^p < 0.01, ^***^p < 0.001. BRE, Bodily Regulation Experience, PRE, Psychological Regulation Experience, SE, Self-Efficacy, ML, Meaning in Life.

#### 6.3.3 Mediating pathway of health Qigong psychological regulation experience on meaning in life

Table 10 shows that, after controlling for all demographic variables, the direct association between psychological regulation experience and meaning in life was statistically significant (*β* = 0.204, t = 4.664, *p* < 0.01). Regarding the indirect pathways, psychological regulation experience was significantly and positively associated with optimism (*β* = 0.467, t = 11.846, *p* < 0.01), and optimism was significantly and positively associated with meaning in life (*β* = 0.106, t = 2.606, *p* < 0.01). Following the same pattern, psychological regulation experience was significantly and positively associated with resilience (*β* = 0.642, t = 17.301, *p* < 0.01), and resilience was significantly and positively associated with meaning in life (*β* = 0.362, t = 8.574, *p* < 0.01). Likewise, psychological regulation experience was significantly and positively associated with hope (*β* = 0.526, t = 14.443, *p* < 0.01), and hope was significantly and positively associated with meaning in life (*β* = 0.143, t = 3.023, *p* < 0.01). Psychological regulation experience was also significantly and positively associated with self-efficacy (*β* = 0.481, t = 12.01, *p* < 0.01). However, the association between self-efficacy and meaning in life was not statistically significant (*β* = 0.066, t = 1.664, *p* > 0.05); the mediating role of self-efficacy was further examined using a bootstrap 95% confidence interval.

The results presented in Table 11 show that, for the model including self-efficacy, the indirect pathway value was 0.028 (*p* = 0.271 > 0.05), with a bootstrap 95% CI of [−0.024, 0.076]. The confidence interval included zero; the indirect pathway was not statistically significant, and no mediating role of self-efficacy was observed between psychological regulation experience and meaning in life. Hypothesis H4a was not supported. For the model including optimism, the indirect pathway value was 0.045 (*p* = 0.017 < 0.05), with a bootstrap 95% CI of [0.009, 0.082]. The confidence interval excluded zero; the indirect pathway was statistically significant. A partially mediating role of optimism between psychological regulation experience and meaning in life was observed, supporting Hypothesis H4b. For the model including resilience, the indirect pathway value was 0.205 (*p* < 0.001), with a bootstrap 95% CI of [0.139, 0.269]. The confidence interval excluded zero; the indirect pathway was statistically significant. A partially mediating role of resilience between psychological regulation experience and meaning in life was observed, supporting Hypothesis H4c. For the model including hope, the indirect pathway value was 0.066 (*p* = 0.008 < 0.01), with a bootstrap 95% CI of [0.018, 0.115]. The confidence interval excluded zero; the indirect pathway was statistically significant. A partially mediating role of hope between psychological regulation experience and meaning in life was observed, supporting Hypothesis H4d.

## 7 Discussion

### 7.1 Relationship between bodily regulation experience and meaning in life

The results indicated that Health Qigong bodily regulation experience was significantly and positively associated with older adults’ meaning in life (*β* = 0.532, *p* < 0.01), supporting hypothesis H1. This finding resonates with recent studies in geriatric physical and mental health (8, 17). Specifically, older adults who consistently practice Health Qigong exhibit a stronger sense of meaning in life, a pattern closely linked to the positive mind–body interactions that unfold during exercise. According to embodied cognition theory, bodily regulation experience restores the body’s subjective status in older adults, thereby establishing their perception of meaning in life (36). As a typical embodied practice, Health Qigong centers on integrating movement, breathing, and mental focus.

First, from the perspective of body perception, the slow, gentle, and continuous movements of Health Qigong direct older adults’ attention to the proprioceptive sensations of various body parts. This clear awareness of bodily boundaries helps to counteract the sense of physical “loss of control” brought about by aging, thereby repairing and reconstructing the perception of continuity in self-existence. As Qu et al. (37) noted, long-term participation in mind–body practices such as Tai Chi significantly enhanced older adults’ body awareness, and this enhanced bodily self-efficacy further translated into more positive evaluations of their overall meaning in life.

Second, from the perspective of the interaction between movement forms and psychological representations, the movements in Health Qigong, such as expanding, opening and closing, and lifting and lowering, are associated with positive psychological representations. For example, “expanding” symbolizes inclusiveness and acceptance, “closing” represents introspection and accumulation, and “lifting” signifies hope and upward striving. When older adults repeatedly perform these body schemas, the corresponding psychological schemas activate simultaneously, allowing the abstract meaning in life to become externalized through concrete physical movements. A neurophysiological study by Wan et al. (38) also found that regular Health Qigong practice enhanced neuroplasticity in brain regions responsible for body representation and interoception, such as the parietal lobe and insula in older adults, providing physiological evidence for the transformation of bodily experiences into positive psychological states.

Finally, deep, slow, and even abdominal breathing can activate the parasympathetic nervous system, reducing older adults’ physiological arousal and anxiety. Within such a calm bodily state, preoccupation with trivial daily concerns tends to recede, and a greater inclination to contemplate and integrate the overall value of life tends to emerge. Dong et al. (39) found that breathing training significantly improved older adults’ attention and emotional state while reducing stress. This effect is achieved by altering the cognitive appraisal of interoceptive signals. Whether the bodily regulation experience identified in this study extends to other mind–body practices, such as yoga and meditation, remains an open question for cross-paradigm comparative research. If systematic differences emerge across practice forms, such a pattern would raise the possibility that the integration of the three regulations reflects a defining core unique to Health Qigong.

### 7.2 Relationship between psychological regulation experience and meaning in life

The results indicated that Health Qigong psychological regulation experience was significantly and positively associated with older adults’ meaning in life (*β* = 0.593, *p* < 0.01), supporting hypothesis H3. This finding aligns with James et al. (40), who reported that Tai Chi and Health Qigong interventions promoted mental health in older breast cancer patients. It affirms the positive role of psychological regulation in older adults’ emotions, cognition, and meaning in life. Health Qigong psychological regulation experience comprises focused attentional guidance, active emotional adjustment, and internal state awareness. It is essentially an embodied practice of mind–body unity.

Firstly, this experience depends on changes in physiological state. Practitioners actively focus attention on specific body parts or an overall sensation of qi while coordinating movements with breathing. This focused attention activates the parasympathetic nervous system and reduces cortisol levels, creating a calm internal state. In this embodied state, older adults detach from everyday existential anxiety and attain a transcendent experience of being. Klussman et al. (41) noted that the mindfulness cultivated through mind–body practices primarily enhanced meaning in life by altering interoceptive states, enabling practitioners to more fully perceive their present-moment experience.

Second, improvements in emotional state during psychological regulation are linked to a shift in individuals’ cognitive appraisal of self and world, alongside changes in bodily sensation. When older adults experience positive emotions and reductions in anxiety and depression through Health Qigong practice, these emotional changes are accompanied by alterations in body expression, breathing rhythm, and muscle tension, extending beyond the psychological level to a new habitual bodily pattern. This positive bodily pattern finds expression in daily meaning-making and is linked to a greater inclination to face later-life challenges with openness and acceptance. De et al. (42) demonstrated that improved emotion regulation in older adults engaged in long-term mind–body exercise significantly enhanced their cognitive appraisal of life coherence and purpose.

Finally, the attentional focus and inner awareness cultivated in psychological regulation experience constitute a refined processing of bodily signals. As older adults repeatedly focus attention on breathing, bodily sensations, and internal states, the insula and anterior cingulate cortex, the brain regions responsible for interoceptive processing, undergo functional remodeling. This neural change enables individuals to identify their internal needs and value orientations with greater clarity and precision (43). Oberoi et al. (44) found that interoceptive awareness cultivated through mindfulness, Tai Chi, and Qigong significantly enhanced individuals’ integration of existential meaning, a neural mechanism underpinned by strengthened functional connectivity between the default mode network and the insula. Whether the psychological regulation experience identified in this study extends to other mind–body practices, such as mindfulness and Tai Chi, remains an open question for cross-paradigm comparative research. If systematic differences in the structure and effects of psychological regulation experience emerge across practice forms, such a pattern would suggest that the guided attention and conscious emotion regulation cultivated in Health Qigong operate in ways distinct from other practice modalities.

### 7.3 Mediating role of psychological capital between bodily regulation experience and meaning in life

The results of this study indicated that optimism, resilience, and hope, respectively, played a partially mediating role in the relationship between Health Qigong bodily regulation experience and older adults’ meaning in life (*β* = 0.048, Bootstrap 95% CI [0.012, 0.086]; *β* = 0.211, Bootstrap 95% CI [0.138, 0.282]; *β* = 0.076, Bootstrap 95% CI [0.028, 0.130]), lending support to hypotheses H2b, H2c, and H2d, while hypothesis H2a was not supported (*β* = 0.025, Bootstrap 95% CI [−0.046, 0.091]). These findings are consistent with the results of Gan et al. (45). According to embodied cognition theory, individuals’ psychological traits are shaped, consolidated, and expressed through continuous interactions between the body and the environment (46). As a highly structured bodily practice, the Health Qigong bodily regulation experience provides an embodied pathway for reconstructing psychological capital.

Firstly, the bodily regulation experience of Health Qigong is linked to gains in hope and optimism within psychological capital, arising from a process-oriented mode of physical training. Health Qigong emphasizes focused attention on, and experience of, the movement process itself. When older adults learn to shift their attention from the measurement of movement standards to the coordination of movements and breathing during practice, this process-oriented bodily attitude is accompanied by the gradual emergence of a cognitive stance: the value of life lies in engagement with the present process rather than anxiety about uncertain outcomes (7).

Secondly, Health Qigong bodily regulation experience is linked to resilience within psychological capital, a quality that emerges alongside the integration of hardness and softness in physical expression. The movement design emphasizes the interplay of movement and stillness, requiring practitioners to use breathing and mental focus to achieve localized relaxation and overall coordination when experiencing physical tension or discomfort, rather than forcibly opposing it. Through repeated practice, this “overcoming hardness with softness” bodily coping mode is accompanied by the internalization of a resilient psychological schema: when facing pressure or setbacks, older adults no longer resort to rigid resistance or avoidance but flexibly adjust their state to seek balance and recovery (47). In this way, the psychological capital elements linked to bodily practice correspond to internal resources for meaning construction, forming a bridge between Health Qigong bodily regulation experience and older adults’ meaning in life.

The absence of a mediating role for self-efficacy between bodily regulation experience and meaning in life may be attributable to two factors. First, the physical improvements associated with bodily regulation experience are highly tangible. When older adults genuinely perceive gains in immunity and physical functioning through practice, these palpable bodily benefits can relate directly to the pursuit of meaning in life, without self-efficacy as a necessary mediator. Second, self-efficacy concerns an individual’s confidence in attaining specific goals, a belief that builds on the prolonged accumulation of mastery experiences rather than short-term acquisition (48). The older adults sampled in this study predominantly approached Health Qigong with the motivation of “nurturing the body and following the natural course” rather than seeking competitive achievement. This cultural orientation stands in tension with the defining characteristics of self-efficacy, namely goal-directedness and measurable success standards: the former privileges process engagement and attunement to bodily experience, whereas the latter rests upon clear outcome expectations and an accrual of mastery experiences. Within this cultural milieu, the conditions conducive to self-efficacy formation, including well-defined task goals and quantifiable success criteria, are not easily met through Health Qigong practice. By contrast, hope (fostered through the gradual improvement of physical functioning and the positive expectations this engenders), optimism (internalized through repeated episodes of bodily comfort into a positive attributional style), and resilience (acquired through a bodily coping mode of overcoming hardness with softness) correspond more naturally to the cultural-psychological disposition of “valuing process over outcome” characteristic of older Chinese practitioners. Accordingly, within this cultural context, Health Qigong practice was less strongly associated with self-efficacy than with other psychological capital dimensions among older adults, and this mediating pathway was not supported.

### 7.4 Mediating role of psychological capital between psychological regulation experience and meaning in life

The results of this study indicated that optimism, resilience, and hope, respectively, played a partially mediating role in the relationship between Health Qigong psychological regulation experience and older adults’ meaning in life (*β* = 0.045, Bootstrap 95% CI [0.009, 0.082]; *β* = 0.205, Bootstrap 95% CI [0.139, 0.269]; *β* = 0.066, Bootstrap 95% CI [0.018, 0.115]), lending support to hypotheses H4b, H4c, and H4d, while hypothesis H4a was not supported (*β* = 0.028, Bootstrap 95% CI [−0.024, 0.076]). These findings are consistent with Afrashteh et al. (49). The mediating roles of optimism, hope, and resilience point to the distinct pathways through which psychological regulation experience is linked to meaning in life.

First, the mediating mechanism of optimism is linked to the repeated reinforcement, within psychological regulation experience, of the perception that one’s physical state can improve. When older adults experience emotional improvement through deep breathing and attentional guidance during Health Qigong practice, this positive bodily transformation is accompanied by the gradual emergence of an optimistic mindset. This mindset is expressed as positive expectations about the overall value of life (50).

Second, the mediating role of hope primarily operates through repeated validation of the “internal intention–bodily response” link. The attentional guidance in Health Qigong is essentially an internal dialogue. When practitioners repeatedly recite mental cues such as “upright and relaxed, natural breathing, focused mind, and concentrating on the lower abdomen” and their bodies indeed relax accordingly, the belief is established at the bodily level: internal effort can bring about real change. This belief, rooted in bodily experience, can rekindle hope in life and activate one’s life value (51). Finally, resilience, as an inner capacity to recover from adversity, benefits directly from the emotion- regulation training inherent in psychological regulation experience, rather than from the suppression of negative emotions. Health Qigong guides practitioners to dissolve them through the coordinated practice of “body, breath, and mind”. When older adults repeatedly experience that negative emotions such as anxiety can be calmed and released, this bodily experience of acceptance-to-transformation becomes internalized as resilience: the ability to accept the present situation, confront setbacks, and seek flexible transformation. This enduring belief and tenacity help older adults in perceiving the continuity and extensibility of life in their daily existence, providing lasting spiritual support (52).

The absence of a mediating role for self-efficacy between psychological regulation experience and meaning in life reflects their essential divergence. Self-efficacy refers to an individual’s belief in their ability to complete specific tasks, a construct whose formation relies on clear behavioral goals and external standards (53). Conversely, the psychological regulation experience of Health Qigong emphasizes release from attachment to outcomes and sustained attention to the practice experience. This orientation toward process engagement is fundamentally at odds with the goal-directed nature on which self-efficacy depends. Psychological regulation experience is thus associated with a process-oriented rather than outcome-oriented psychological resource, and self-efficacy, given its inherent goal-directedness, is unlikely to mediate this pathway.

This tension becomes particularly salient in the Chinese cultural context. The core principles of psychological regulation experience, namely focused attention, emotional calm, and inner tranquility, find a deep resonance with the traditional Chinese health-preserving philosophy encapsulated in the classical dictum “tranquil emptiness, spirit guarded within”, a mode of self-cultivation oriented toward inward seeking drawn from the *Huangdi Neijing*. This mode of cultivation does not presuppose the attainment of external benchmarks; it pursues an inner state of mind–body unity as an end in itself. By contrast, the social-cognitive foundation of self-efficacy, rooted in Bandura’s account of building competence beliefs through successful experiences, presupposes external standards against which performance can be evaluated. In the Health Qigong practice of Chinese older adults, the sense of mind–body unity brought about by psychological regulation experience constitutes the purpose of practice, rather than serving as a means to an external goal. When practitioners arrive at inner calm through deep breathing and guided intention, the value of this experience resides in life itself, without a need to anchor life’s meaning in confidence about completing a particular task.

### 7.5 Research implications

#### 7.5.1 Theoretical implications

First, this study developed the Health Qigong Embodied Experience Scale through qualitative methods, coding its dimensions and validating the scale’s feasibility through quantitative procedures, before constructing a mediation model to examine the associations among Health Qigong bodily regulation experience, psychological regulation experience, and older adults’ meaning in life. This progression moves embodied cognition theory from qualitative exploration into quantitative testing, thereby establishing an empirical framework that can be tested and extended in future research.

Second, this study introduced COR theory as a macro-level explanatory framework for the mediating mechanism of psychological capital, constructing a “resource gain → resource accumulation → resource mobilization” logic chain connecting Health Qigong embodied experience to meaning in life. At the macro level, COR theory delineates the structural architecture of the mediating pathways; at the micro level, embodied cognition theory accounts for how bodily regulation experience and psychological regulation experience relate to meaning in life. These two theoretical lenses operate at complementary levels of analysis and jointly enrich the theoretical foundations of this research domain.

Third, the parallel mediation and independent model designs revealed differentiated mediating roles across the four psychological capital dimensions and the two types of embodied experience. Hope, resilience, and optimism each carry distinct mediating functions, whereas the mediating role of self-efficacy was not supported. This finding may reflect the cultural and regional characteristics of the present sample, which was drawn from older adults in Guangzhou, China. Within the traditional Chinese cultural context, Health Qigong is understood as a gentle practice of self-cultivation rather than a competitive pursuit, and older practitioners predominantly approach it with the guiding orientation of “nurturing the body and following the natural course.” This cultural orientation stands in tension with the defining features of self-efficacy, namely goal-directedness and the accumulation of mastery experiences: the former privileges process engagement, whereas the latter requires outcome attainment. Within this cultural milieu, Health Qigong practice thus bears a weaker association with self-efficacy than with other psychological capital dimensions among older adults. This pattern, which distinguishes the present study from prior work, offers a more contextually sensitive theoretical perspective for subsequent research.

Fourth, the development of the Health Qigong Embodied Experience Scale fills a notable measurement gap in the field and holds important scientific significance. Most existing studies have treated Health Qigong as an intervention tool, focusing on whether it is effective while providing no standardized assessment of what practitioners actually experience during practice. Grounded in embodied cognition theory, the present scale extracted two core dimensions, bodily regulation experience and psychological regulation experience, through qualitative coding of interviews with older adults and experts. Its psychometric properties were established through a two-stage validation comprising preliminary testing and a formal survey. The scientific value of this scale resides in its translation of abstract embodied cognition concepts into quantifiable measurement indicators, offering a foundation for future investigations into the relationships between Health Qigong embodied experience and broader mental health outcomes. Moreover, it enables a wider range of audiences to perceive the health benefits of Health Qigong through the concrete dimensions of bodily regulation experience and psychological regulation experience.

#### 7.5.2 Practical implications

First, the application of the measurement tool. Comprising only 10 items and requiring minimal completion time, the present scale is well suited for large-scale administration among older adult populations and highly practical in field settings. Researchers may deploy the scale flexibly according to their specific objectives: the bodily regulation experience subscale can be used independently when the focus is on changes in physical functioning perception, the psychological regulation experience subscale can be used independently when the focus is on changes in psychological states, and the two subscales can be administered jointly when a comprehensive assessment of embodied experience is needed. Beyond this flexibility, the scale’s practical value lies in its capacity to serve as a common measurement tool for interdisciplinary collaboration. Sports science researchers may use it to quantify the effects of movement instruction, psychology researchers may use it to track associations between bodily experience and psychological indicators, and medical researchers may use it to evaluate improvements in the embodied dimension of rehabilitation interventions. With three disciplines converging on the same instrument, disciplinary barriers become easier to bridge, helping to move Health Qigong research from single-discipline inquiry toward genuine interdisciplinary integration. Furthermore, the process-oriented design of the scale supports repeated administration at multiple time points in intervention studies, enabling researchers to track dynamic changes in embodied experience across the practice period. This yields process-level data that can inform the phased refinement of experimental protocols and guide adjustments to intervention strategies in response to shifts in practitioners’ embodied experience.

Second, the refinement of instructional practice. Building on the present findings, Health Qigong instructors are encouraged to attend to learners’ embodied sensations alongside movement accuracy. The bodily regulation experience dimension calls for instruction that anchors learners’ attention in sensations of bodily extension, deep and prolonged breathing, and the smooth circulation of qi and blood. The psychological regulation experience dimension calls for attentional guidance and emotion- regulation components, supporting learners in experiencing inner calm during practice. To cultivate psychological capital among older adults, instruction should center on hope, optimism, and resilience as primary intervention targets. Hope may be cultivated through progressive advancement from isolated slow practice to combined movement sequences, enabling older adults to build positive expectations through the ongoing experience of gradual improvement. Optimism may be strengthened by guiding learners to attend closely to their immediate mind–body sensations at the close of each session. Resilience may be enhanced through practice components that.

teach the release of tension via breathwork, equipping older adults with a coping style rooted in gentle dissolution rather than rigid resistance. Notably, cultivating these three resources does not require high-intensity training or standardized skill attainment; it unfolds naturally through low-intensity, experience-centered practice.

Third, community health promotion. The present findings offer an evidence-based reference for the community-level promotion of Health Qigong in the context of active aging. Compared with intervention models oriented toward competency enhancement and goal attainment, Health Qigong programs centered on optimizing process experience have a lower cultural threshold and are better suited to older adult populations. The mediating pathways identified in this study provide community health workers with concrete intervention targets for designing active aging programs: taking the optimization of older adults’ embodied experience as the point of entry may facilitate the activation of internal psychological resources, which may in turn contribute to enhancing their meaning in life. This pathway does not require older adults to meet specified skill benchmarks; it asks only for sustained participation and mindful engagement, allowing physical and psychological benefits to emerge through the practice process itself. For large-scale community implementation, this combination of low entry barriers and high population fit offers distinct practical advantages, providing an actionable route for translating active aging from a policy vision into community practice.

### 7.6 Limitations and future directions

First, cross-sectional design. The mediation analyses in this study used cross-sectional data collected at a single time point. The mediation model and bootstrap method were applied to reveal the association structure among variables, rather than to assert causal direction. This pattern of associations provides well-defined hypothetical pathways for causal testing in future longitudinal research. Future research could adopt experimental intervention and longitudinal designs, collecting data at multiple time points while manipulating key variables, to further examine the robustness of the mediation pathways identified in this study and clarify their underlying causal mechanisms.

Second, sampling method. This study employed convenience sampling, with participants drawn from older adults across six communities in Guangzhou. Owing to the non-probability nature of convenience sampling, the sample is geographically concentrated and does not represent the broader older adult population, limiting the generalizability of the findings. Whether the current conclusions extend to older adults in other regions warrants further examination. Future studies should survey older adult participants from a wider range of geographic areas and adopt stratified or quota sampling methods to enhance geographic representativeness and further examine the generalizability of the findings. Furthermore, future cross-cultural comparative research would help assess the extent to which the present findings generalize to older adult populations in different cultural contexts.

Third, self- reports. All data in this study were obtained through older adults’ self-reports, which may be susceptible to common method bias and social desirability effects. Although Harman’s single-factor test yielded results within the acceptable range, future research could incorporate multiple complementary methods for cross-validation, including behavioral observation, physiological indicators, and informant reports.

Fourth, the qualitative research method requires further refinement. The qualitative component of this study would benefit from greater rigor in dimension extraction and coding procedures. Future research should employ grounded theory to code the scale dimensions step by step, thereby strengthening the consistency and rigor of the scale’s internal structure.

Fifth, the target population of the newly developed scale should be broadened. The scale has undergone preliminary validation only among older adults. Future research should extend its application to other groups, such as university students and adolescents, to further examine the scale’s applicability. Feedback from these groups will inform continued optimization of the scale, facilitating its application across diverse psychological mechanism studies.

## 8 Conclusion

This study consisted of qualitative and quantitative components. In the qualitative component, thematic analysis was used to code the dimensions of the new scale, followed by the development and validation of the Health Qigong Embodied Experience Scale through statistical methods. In the quantitative component, COR theory framed the structural relationships among the mediation pathways at the macro level, while embodied cognition theory was applied at the micro level to discuss the associations between Health Qigong embodied experience and older adults’ meaning in life. The mediating roles of the dimensions of psychological capital in the relationships between Health Qigong embodied experience and older adults’ meaning in life were also discussed. The findings indicated that Health Qigong bodily regulation experience and psychological regulation experience were each associated with older adults’ meaning in life. Furthermore, optimism, resilience, and hope each partially mediated the relationships between Health Qigong bodily regulation experience, psychological regulation experience, and older adults’ meaning in life. A further finding indicated that self-efficacy did not mediate these relationships, a noteworthy finding of this study.

## Data Availability

The raw data supporting the conclusions of this article will be made available by the authors, without undue reservation.
